# *Aspergillus* Goes Viral: Ecological Insights from the Geographical Distribution of the Mycovirome within an *Aspergillus flavus* Population and Its Possible Correlation with Aflatoxin Biosynthesis

**DOI:** 10.3390/jof7100833

**Published:** 2021-10-05

**Authors:** Francesca Degola, Giorgio Spadola, Marco Forgia, Massimo Turina, Lucia Dramis, Walter Chitarra, Luca Nerva

**Affiliations:** 1 Department of Chemistry, Life Sciences and Environmental Sustainability, University of Parma, Parco Area delle Scienze 11/A, 43124 Parma, Italy; giorgio.spadola1@unipr.it (G.S.); lucia.dramis@unipr.it (L.D.); 2 Institute for Sustainable Plant Protection, CNR, Strada delle Cacce 73, 10135 Torino, Italy; marco.forgia@ipsp.cnr.it (M.F.); massimo.turina@ipsp.cnr.it (M.T.); walter.chitarra@crea.gov.it (W.C.); 3 Council for Agricultural Research and Economics—Research Centre for Viticulture and Enology CREA-VE, Via XXVIII Aprile 26, 31015 Conegliano, Italy

**Keywords:** *Aspergillus flavus*, mycovirome, aflatoxin, vivivirus, fungal population ecology

## Abstract

Microbial multi-level interactions are essential to control the success of spreading and survival of most microbes in natural environments. Phytopathogenic mycotoxigenic fungal species, such as *Aspergillus flavus*, represent an important issue in food safety. Usually, non-toxigenic strains are exploited for biocontrol strategies to mitigate infections by toxigenic strains. To comprehend all the biological variables involved in the aflatoxin biosynthesis, and to possibly evaluate the interplay between *A. flavus* toxigenic and non-toxigenic strains during intraspecific biocompetition, the “virological” perspective should be considered. For these reasons, investigations on mycoviruses associated to *A. flavus* populations inhabiting specific agroecosystems are highly desirable. Here, we provide the first accurate characterization of the novel mycovirome identified within an *A. flavus* wild population colonizing the maize fields of northern Italy: a selection of *A. flavus* strains was biologically characterized and subjected to RNAseq analysis, revealing new mycoviruses and a peculiar geographic pattern distribution in addition to a 20% rate of infection. More interestingly, a negative correlation between viral infection and aflatoxin production was found. Results significantly expanded the limited existent data about mycoviruses in wild *A. flavus*, opening new and intriguing hypotheses about the ecological significance of mycoviruses.

## 1. Introduction

As important members of the microbial community, the interactions of fungi with other organisms variously influence different aspects of both agriculture and human health [1,2,3]. The benefits of fungi/plant associations are well-known. For instance, the improvement of abiotic and biotic stresses’ tolerance of the host, as well as its mineral nutrition, is ascribed to the presence of symbiotic fungi [4,5,6]. On the other hand, fungal pathogenic interactions are also well-known, and highly undesirable, as in the case of phytopathogenic species infesting economically relevant crops [7]. Since the first characterization of *Cryphonectria hypovirus 1* (CHV1), it became clear that mycoviruses have relevant effects on the host behavior [8]. Indeed, during the last decades, some biological processes, including virulence and sporulation, which are fundamental for both the colonization of the environment and the ecological fitness of the fungus, were demonstrated to be influenced by the presence of mycoviruses in many fungi. Attenuation of virulence, alteration of colonies’ morphology, reduction of conidia production, modification of accumulation of secondary metabolites (SMs), and decrease of growth rate in their hosts are the most commonly detected negative effects [9,10,11,12,13]. Although the role of fungi-associated viruses still remains largely unknown and the relevant literature is scarce in comparison to the number of papers produced on the plant viruses, their capacity in attenuating the pathogenicity of the fungal host and/or in modulating the biosynthesis of toxic metabolites is of great interest to the scientific community. Some mycoviruses in fact could be considered detrimental when they attack edible and/or commercial mushrooms, but their potential use as biological control agents against fungal phytopathogens in economically important plants has been recently explored. Several approaches relying on the application of hypo-virulent strains have been documented, aimed at the management of common fungal diseases in plants [14,15,16,17]. To date, the high variability in terms of efficacy has been attributed to some biological barriers, such as vegetative incompatibility [18,19], which is hence expected to be a key factor that might limit the wide exploitation of mycoviruses in agricultural programs. This data on mycoviruses suggests, in turn, that the development of virus-based antifungal products requires to consider multiple factors, including both host and virus biological features [20]. Despite these drawbacks, the discovery and the study of an increasing number of novel mycoviruses have undoubtedly widened our knowledge about their ecology and evolution, comprehensively improving our understanding of viral diversity among fungal species, their phylogenetic relationships, and their interactions with the environment [21,22]. Amongst the fungal genera, *Aspergillus* is probably the most studied, because of its agricultural, clinical, and economic implications: the first mycoviruses reported in *Aspergillus* species dates back to 1970 [23], but to date, viral particles in strains belonging to *Nigri*, *Clavati*, *Circumdati*, *Fumigati,* and *Flavi* sections have been described ([14] and references therein). The interaction between mycoviruses and mycotoxigenic fungi—particularly aflatoxigenic species—has been debated since 1986, when the effects of some viruses on the aflatoxin (AF) biosynthesis in *A. flavus* were reported for the first time [24]. More recently, it has been reported that transfection of the complete double-stranded RNA linear genome segments (dsRNA) of the Penicillium chrysogenum virus (PcV) in a toxigenic *A. flavus* strain resulted in a stable suppression of aflatoxin biosynthesis. This was probably due to RNA interference (RNAi) mechanisms specifically directed against AF-related gene transcripts as a result of a structural similarity shared between viral dsRNA of non-toxigenic strains and AF-related genes [25,26]. Interaction studies on mycotoxigenic species and mycoviruses belonging to the *Partitiviridae* family shed light on a possible role of some viruses in mycotoxin regulation, suggesting significant influences on the biocontrol potential exerted by some fungal hosts [12,13,27,28]. This is particularly interesting if we consider that a strategy of intraspecific biocompetition for the control of AF contamination in various crops is based on the use of non-toxigenic strains as biological control agents (BCA) [29]. However, the mechanisms of both non-toxigenicity and biocompetition have been only partially clarified for these strains. Aflatoxin-producing strains were likely to be infected by dsRNA as non-producing strains, and, furthermore, the eradication of viruses from infected strains did not result in a loss of their toxigenic ability [30,31,32]. Nonetheless, due to the high occurrence of *A. flavus* in the agroecosystem, particularly on cereal crops, and the associated repercussions for human and animal health in terms of harmful AF contamination of feed and food commodities, the biological importance of any interaction with mycoviruses deserves to be investigated in more detail. In Italy, there are more than 1 million hectares dedicated to maize cultivation, which are located mainly in northern Italy and which account for 16 million tons of final products (https://ec.europa.eu/eurostat/data, accessed on 21 May 2020). Since 2003, due to the ongoing climate change scenario, maize cultivation in northern Italy has started to suffer from high aflatoxin contamination, which is favored by both humid-hot summers and plant water stress [26]. Therefore, an urgent need exists for new control strategies of *A. flavus* spread and aflatoxin diffusion. However, despite the great potential of mycoviruses in the arsenal of biocontrol fungal agents, no accurate characterization of the virome of a wild *A. flavus* population has yet been carried out. The present work is aimed at investigating and describing, for the first time, the mycoviruses represented within an *A. flavus* wild population that colonize the maize fields of northern Italy.

## 2. Materials and Methods

### 2.1. Fungal Isolates and Growth Conditions

*A. flavus* strains were isolated from maize kernels from six regions of northern Italy, namely Piemonte (Pie), Emilia Romagna (Emi), Trentino Alto-Adige (Taa), Lombardia (Lom), Veneto (Ven), and Friuli Venezia-Giulia (Fri). Isolates were grown and maintained on YES agar medium (20 g/L granulated yeast extract, 50 g/L sucrose, 20 g/L agar) at 28 °C. All the strains are deposited in the laboratory collection (University of Parma) and available upon direct request to the authors. For gDNA analysis, mycelium cultured in YES liquid medium for 6 days at 28 °C, under stationary conditions, was used. For total fungal and viral RNA extraction, mycelium was obtained from conidial inocula cultured in YES liquid medium at 28 °C, in an orbital shaker at 110 rpm for 4 days. 

### 2.2. Chemotype Characterization of A. flavus Strains

The AF production ability of isolates was assessed in standard flat-bottom 96-well microplate (Sarstedt, Newton, NC, USA) cultures, as previously described [33]. Coconut clarified medium was used, and plates were incubated in the dark under stationary conditions for 6 days at 25 °C. The detection of aflatoxin released in the medium was performed with a fluorescence microplate reader (TECAN SpectraFluor Plus, Männedorf, Switzerland) using the following parameters: λ_ex_ = 360 nm, λ_em_ = 465 nm, manual gain = 83, lag time = 0 µs, number of flashes = 3, and integration time = 200 µs. Each strain was analyzed in four biological replicates, and analysis for each replicate was repeated three times.

### 2.3. Sclerotia Biogenesis Evaluation

Point inoculations (5 μL from a 1 × 10^5^ conidia/mL suspension) of each *A. flavus* strain were centrally spotted on Czapek agar plates (1 g/L di-potassium hydrogen phosphate, 0.5 g/L magnesium sulfate heptahydrate, 0.5 g/L potassium chloride, 3 g/L sodium nitrate, 30 g/L sucrose, 15 g/L agar), according to [29]. Cultures were incubated for up to 10 days at 30 °C to induce sclerotia biogenesis in triplicate. Sclerotia diameter was measured for attributing the L/S type, where isolates with sclerotia diameter > 400 µm have been classified as L and isolates with sclerotia diameter < 400 µm have been classified as S.

### 2.4. A. flavus gDNA Extraction and RAPD-PCR Profile Characterization 

Genomic DNA was extracted using DNAzol™ reagent (Life Technologies, Invitrogen, CA, USA), according to the manufacturer’s instructions. Extracted samples were quantified with NanoDrop 2000 (Thermo Fisher Scientific, Waltham, MA, USA), and their quality was checked via electrophoresis on 1% agarose gel.

RAPD-PCR (Random Amplification of Polymorphic DNA) analysis was conducted using a degenerate oligonucleotide (5′-GAGAGAGAGAGAGAGAYG-3′), and 5 ng of gDNA was used as a template for the amplification reaction conducted with the GoTaq^®^ DNA polymerase Kit (Promega, Madison, WI, USA). The reaction was performed in 20 μL of PCR Flexi Buffer 5X (Promega, Madison, WI, USA), 25 mM of MgCl_2_, 25 mM of dNTPs, 10 μM of primer, and 0.5 U of Taq DNA-polymerase. Amplification parameters were: 4 min at 94 °C, for 35 cycles: 1 min at 94 °C, 20 s at 44 °C, and 2 min at 72 °C, and final extension for 6 min at 72 °C. Resulting RAPD-PCR patterns were visualized on 2% agarose gel.

### 2.5. RNA Extraction and Viral Sequences’ Detection

Total RNA was extracted from *A. flavus* mycelium following the TRIzol^®^ protocol (Life Technologies, Invitrogen, Carlsbad, CA, USA). RNA concentration was estimated using NanoDrop 2000 (ThermoFisher Scientific, Carlsbad, CA, USA), while quality was checked on 1% agarose gel. To proceed with RNA-seq analysis, a mixed sample containing all the RNAs in equal amount was prepared, by mixing 500 ng of RNA from each isolate, as previously reported [34]. The pooled sample containing RNA from all the fungal isolates was sent to Macrogen Inc. (Seoul, South Korea) for rRNA depletion (Ribo-Zero™ Gold Kit, Epicentre, Madison, WI USA), cDNA libraries’ construction (TrueSeq total RNA sample kit, Illumina, San Diego, CA, USA), and sequencing by Illumina Novaseq technology with an output of 100 M paired-end reds of 100 bp.

De novo assembly of sequenced RNA was achieved using high-quality and clean sequences selected using Trimmomatic [35]. Trinity (version 2.3.2) was used to assemble the selected cleaned reads [36]. Viral sequences were identified searching for conserved domains with blastx (version 2.6.0+) from the BLAST suite. BWA 0.7.15-r1140 [37] and SAMtools 1.3.1 [38] were used to align the original reads against the assembled viral contigs. Coding open reading frames (ORFs) were detected with ORF Finder (http://www.ncbi.nlm.nih.gov/gorf/orfig.cgi, accessed on 2 July 2021) and then blasted to NCBI nr databases.

### 2.6. Validation of In Silico Detected Viruses 

For each *A. flavus* strain, previously obtained RNA was used as a template for cDNA synthesis (Omniscript RT Kit, QUIAGEN), following the manufacturer’s instructions. Amplification of *A. flavus* β-tubulin and viral RNAs was obtained with iTaq universal SYBR Green super-mix (Bio-Rad) and specific primers (Supplementary Table S1). Cycling parameters were: 2 min at 50 °C, then 10 min at 95 °C, for 40 cycles: 15 s at 95 °C, 1 min at 60 °C, dissociation curve: 15 s at 95 °C, 1 min at 60 °C, and 15 s at 95 °C. Three independent biological replicates were present in each quantification experiment, and three technical replicates for each biological replicate were analyzed in the RT-qPCR.

### 2.7. Virus Particles’ Purification and Electron Microscopy Observation

Three hundred mg of lyophilized mycelia (previously grown in liquid culture) were used for the purification of virus-like particles using a classic differential centrifugation protocol. The MN1 sample was homogenized with sterile glass and iron beads (4 mL of 0.5 mm in diameter glass beads and 1 mL of 1 mm iron beads) using a bead beater in 10 mL of extraction buffer (0.25 M potassium–phosphate pH 7, 10 mM EDTA, 0.5% thioglycolic acid). A first centrifugation at 1000× *g* for 10 min was performed to eliminate cell debris, the supernatant was recovered and transferred into a new sterile 50 mL tube, amended with 1% Triton X-100, 0.5% propanesulfonate detergent, 10% PEG8000, and 0.1% sodium chloride, and stirred for 120 min at 4 °C. Then, the mixture was centrifuged for 15 min at 10,000× *g*. The obtained pellet was resuspended into extraction buffer and centrifuged on a 20% sucrose layer at 180,000× *g* [21,39]. The resulting pellet was resuspended in 300 µL of extraction buffer and subjected to a sucrose gradient (50% to 20% in extraction buffer). After centrifugation, the only visible layer was collected and precipitated by centrifuging at 200,000× *g* for 1 h.

For TEM observation of virus particles, samples were allowed to adsorb on carbon and formvar-coated grids for at least 5 min. After adsorption, grids were washed with water and then negatively stained with a 0.5% solution of uranyl acetate. Observations and image captures were made using a CM 10 electron microscope (Philips, Eindhoven, The Netherlands).

### 2.8. Data Analysis

A hypergeometric-based test was used to assess the significance of correlations across samples between mycovirus infection and AF production. The rationale behind using the hypergeometric test was that if mycoviruses have functional association with the inability to produce AF, they would be found enriched in the subsample of virus-infected isolates. We tested under the null hypothesis that the viral infection in one sample is independent from the AF production within the same sample. The *p*-value was derived from the hypergeometric function, where N represent the population size, M is the total number of AF-producing isolates, s is the subsample of virus-infected isolates, and k is the number of AF-producing isolates in the subsample.

## 3. Results 

### 3.1. Selection of A. flavus Strains and Chemotype/Sclerotia Production Characterization of the Population

*A. flavus* strains were isolated from kernels belonging to northern Italian maize fields and were firstly subjected to random amplified polymorphic DNA (RAPD)-PCR analysis to define their molecular profile. A total of 250 isolates were screened, and strains with different RAPD amplification patterns were selected (an example is provided in Supplementary Figure S1) and assayed for aflatoxin production and sclerotia biogenesis. Amongst them, about 65% have been classified as AF-producers (toxigenic strains), and about 60% have been classified as sclerotia-producing (sclerotigen) strains. 

### 3.2. Mycovirome Characterization

**Figure 1:**
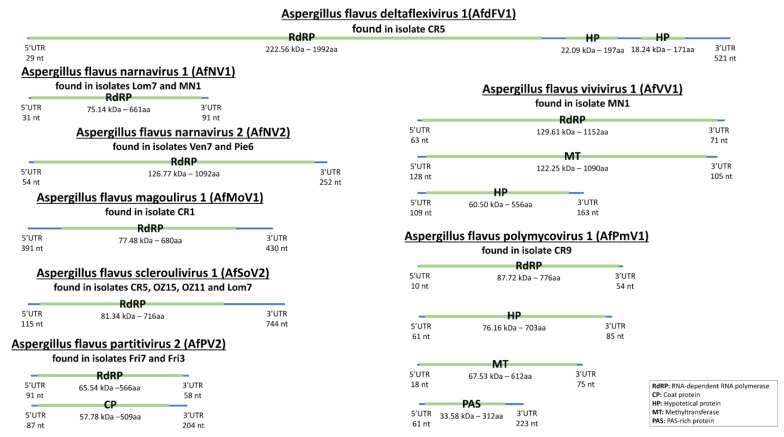
Representation of the genome organization of viruses identified in the present study. In blue are depicted the non-coding segments of genomes, whereas green portions represent the detected ORFs.

A homogeneous sub-population of 62 strains was built by sorting an equal number of aflatoxigenic/non-aflatoxigenic and sclerotia-producing/non-producing isolates, and all strains belonged to the L-type (Table 1). RNA-seq analysis revealed that about 20% of our *A. flavus* population were infected by one or more mycoviruses (Table 2). Among these strains, 4 isolates were infected by more than one virus, and 8 with only one. An overview of the viruses identified in the present work and their genomic organization are reported in Figure 1 and Table 3.

**Figure 2:**
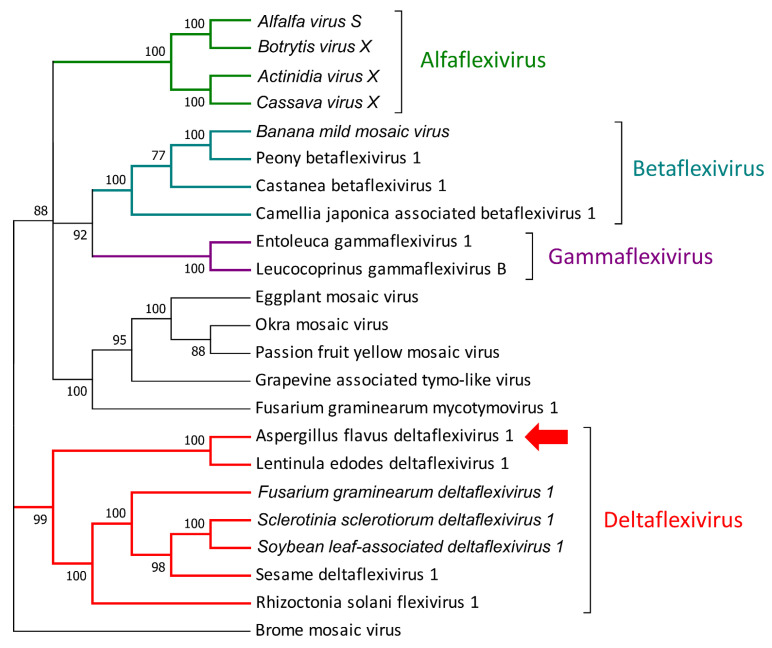
Phylogenetic relationship of the virus belonging to the *Tymovirales* order. Amino acids’ sequences of RNA-dependent RNA polymerases (RdRps) were aligned using MUSCLE, and phylogeny were then derived using the likelihood methodology in IQTREE. Numbers above branches represent statistical support based on bootstrap analysis (1000 replicates). The virus identified in this work is indicated by the colored arrow.

The first identified virus belongs to the Tymovirales order and presents similarities to an undefined virus isolated from soil samples (QDH90368.1) and to Lentinula edodes deltaflexivirus 1 (QOX06047.1): it shows two conserved domains along the first ORF (from nucleotide 29 to nucleotide 6007), the first at the 5′ (about 1 Kb from the first nucleotide), which shows high homology to viral methyltransferases, and the second, which is located about 4 Kb from the first nucleotide, which encodes for a viral helicase. The genome then presents another two ORFs encoding for two proteins with no conserved domain and with no homology with other proteins in NCBI databases. The phylogenetic analysis of the RdRP sequence displayed a good relationship with Lentinula edodes deltaflexivirus 1 (QOX06047.1), confirming its relationship with the *Deltaflexiviridae* family (recognized from year 2019 by the International Committee for Taxonomy of Viruses (ICTV), https://talk.ictvonline.org/, accessed on 2 July 2021), and for this reason, we propose the name *Aspergillus flavus* deltaflexivirus 1 (AfDfV1) (Figure 2). 

**Figure 3:**
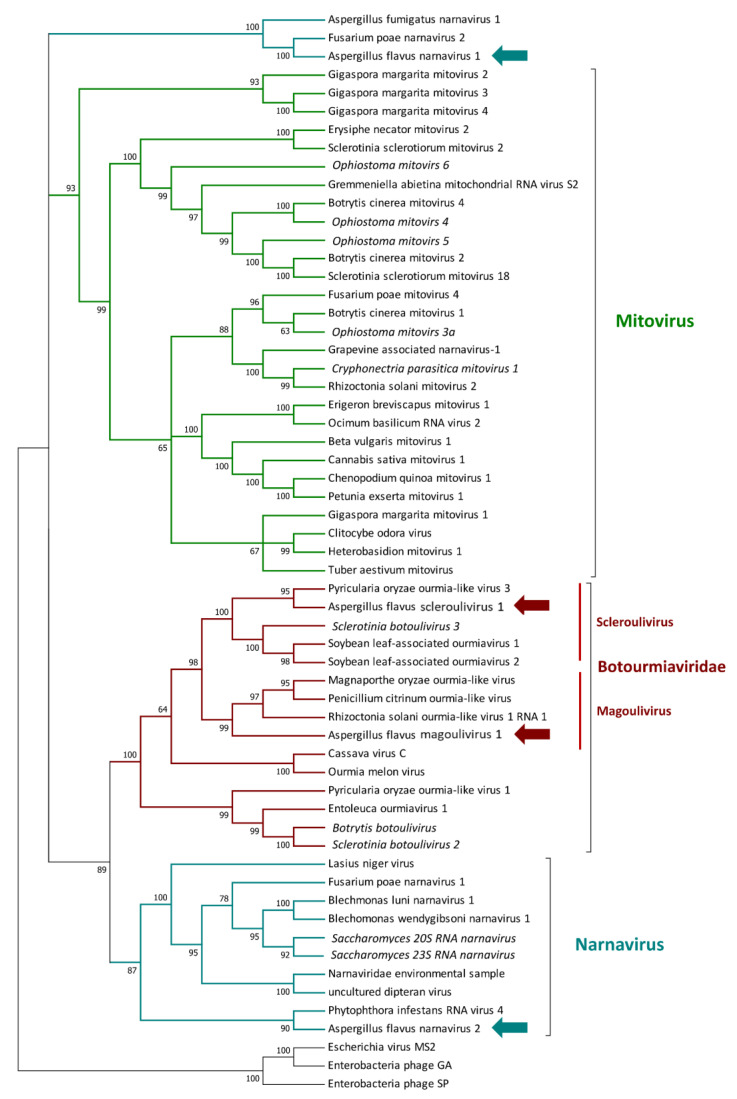
Phylogenetic relationship of viruses belonging to the Lenarviricota phylum. Amino acids’ sequences of RNA-dependent RNA polymerases (RdRps) were aligned using MUSCLE, and phylogeny were then derived using the likelihood methodology in IQTREE. Numbers above branches represent statistical support based on bootstrap analysis (1000 replicates). Viruses identified in this work are indicated by colored arrows.

Two viruses showing homology to the *Narnaviridae* family [40] were identified: the first one presents a three segmented genome, with apparently no conserved domain (as observed by the CDD/SPARCLE analysis) and probably belonging to a new group of narnaviruses defined as splipalmiviruses [41]. The second virus possesses a 3486 nt genome and displays 55.6% identity with Plasmopara viticola lesion-associated narnavirus 32 (QIR30311.1) [42]. The phylogenetic analysis suggested that, despite both viruses being grouped with other narnaviruses, they probably belong to two different clades, as indicated by their different genome size and phylogenetic placement (Figure 3). We propose for the two viruses the names *Aspergillus flavus* narnavirus 1 (AfNV1) and *Aspergillus flavus* narnavirus 2 (AfNV2), respectively. Two other new viruses belonging to the Lenarviricota phylum were found: the first one is a 2863 nucleotides-long segment which displays a 2043 nucleotides ORF encoding a putative RdRP. The protein showed 88.14% identity to Spodoptera exempta virus TenAfr-2017 (QQZ00864.1), but no conserved domain was detected during the CDD/SPARCLE analysis. The second identified viral genome was a 3009 nt long sequence that exhibited a 2151 nucleotides ORF encoding for a putative RdRP, which shares 43.3% identity to Oidiodendron maius ourmia-like virus 1 (QNN89181.1) [41]. The phylogenetic placement of both viruses confirmed that they belong to the *Botourmiaviridae* family [43]: the first was named *Aspergillus flavus* magoulivirus 1 (AfMoV1) due to its phylogenetic relationship with the *Magoulivirus* genus, while the second one was named *Aspergillus flavus* scleroulivirus 1 (AfSoV1), due to its phylogenetic relationship with the *Scleroulivirus* genus (Figure 3).

**Figure 4:**
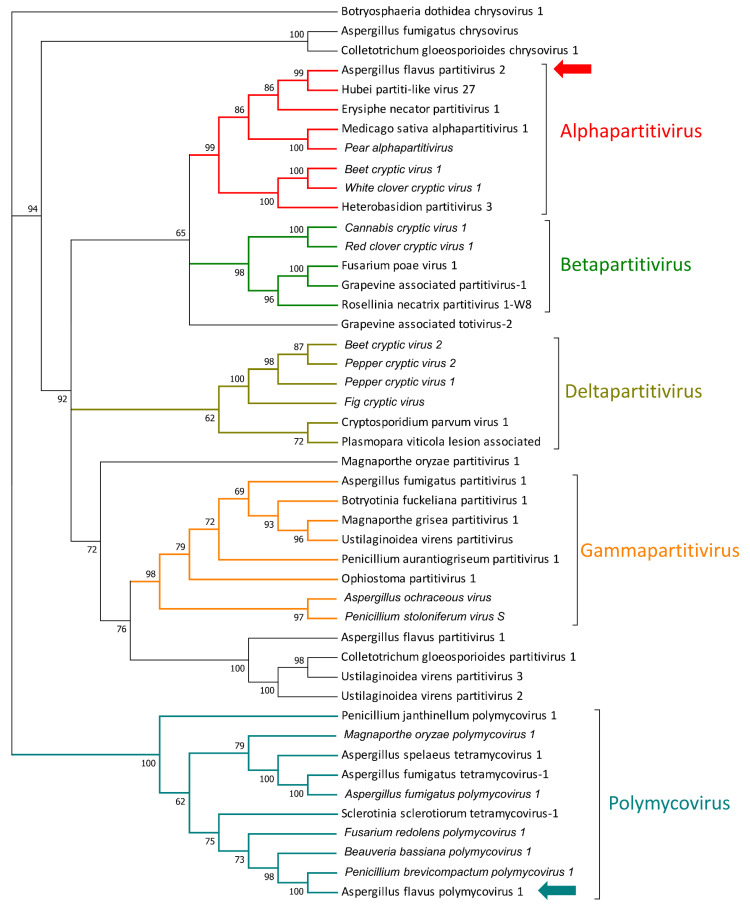
Phylogenetic relationship of viruses displaying a double-stranded RNA (dsRNA) genome. Amino acids’ sequences of RNA-dependent RNA polymerases (RdRps) were aligned using MUSCLE, and phylogeny were then derived using the likelihood methodology in IQTREE. Numbers above branches represent statistical support based on bootstrap analysis (1000 replicates). Viruses identified in this work are indicated by colored arrows.

Additionally, a virus with a bipartite genome was also identified: the first RNA (RNA1) is 1849 nt long and presents a 1701 nt ORF that encodes for a putative RdRP, as also suggested by the CDD/SPARCLE analysis that identified an RT-like domain. The closest proteins are two putative RdRP encoded by Hubei partiti-like virus 27 (APG78241.1) [44] and by Lichen partiti-like RNA virus sp. (BCD56390.1) [45], both displaying an amino acid sequence with 55% identity to the newly identified virus. The second segment (1820 nt long) displays a unique ORF of 1530 nt, which encodes for a protein with no conserved domain, but with some similarities to the putative coat protein of parti-like and partitiviruses. The phylogenetic placement suggested that this virus belongs to the *Partitiviridae* family [46] and, more specifically, to the *Alphapartitivirus* genus (Figure 4); for this reason, we propose the name of *Aspergillus flavus* partitivirus 2 (AfPV2). 

We detected a second virus belonging to the dsRNA clade, which presents a multipartite genome made of 4 segments. The first segment (RNA1) is 2394 nt long (2331 nt length ORF) and codes for a protein with a conserved RT-like domain (cl02808). The second segment (RNA2), encoding a protein with a conserved methyltransferase domain, is 1931 nt long and presents a 1839 nt ORF. The third segment (RNA3—1222 nt in length) displays a 939 nt long ORF, and this ORF did not display any conserved domain, but it is related to several proline/alanine (PAS)-rich proteins of previously reported polymicoviruses. The last segment (RNA4—2257 nt) codes for a protein with no conserved domain and with no predicted function. Due to its similarity to other polymycoviruses and to its phylogenetic placement (Figure 4), we named it *Aspergillus flavus* polymycovirus 1 (AfPMV1).

**Figure 5:**
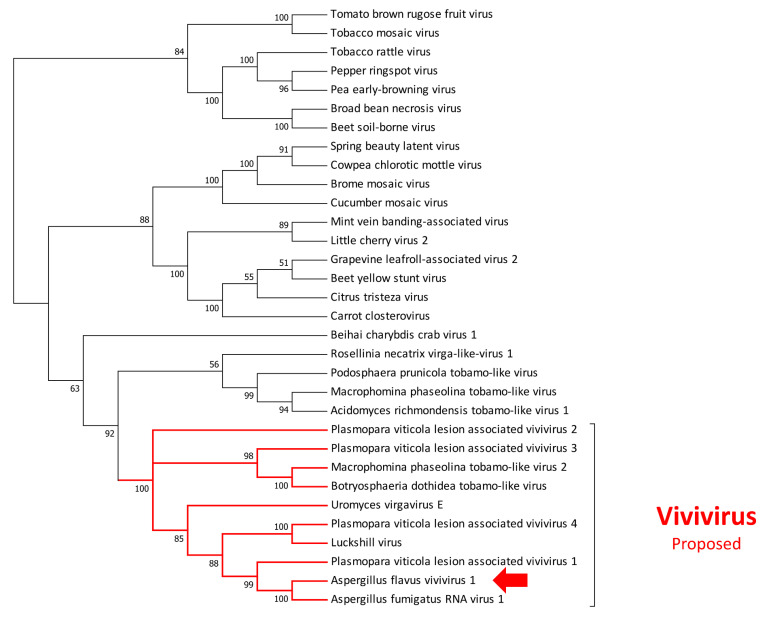
Phylogenetic relationship of the virus belonging to the new proposed genera Vivivirus. Amino acids’ sequences of RNA-dependent RNA polymerases (RdRps) were aligned using MUSCLE, and phylogeny were then derived using the likelihood methodology in IQTREE. Numbers above branches represent statistical support based on bootstrap analysis (1000 replicates). The virus identified in this work is indicated by the colored arrow.

Finally, we identified a virus with three putative segments, the first one (RNA1) is 3592 nt long, displays a 3459 nt long ORF, and putatively codes for a protein with a conserved RdRP domain. The second is 3509 nt long with a 3273 nt long ORF and encodes a protein with two conserved domains: a methyltransferase at the 5′ and a viral helicase at the 3′. The third segment is 1942 nucleotides long, presents a 1671 nt long ORF, and encodes for a protein with no detected conserved domain. All three proteins displayed a relatively good degree of conservation with *Aspergillus fumigatus* RNA virus 1 (txid2747487), a virus recently reported in NCBI [47]. For the first two segments, several other similarities were also observed with a recently reported virus associated with *Plasmopara viticola*. Similarly to what was reported for Plasmopara viticola lesion-associated vivivirus 1 [42], we observed a conserved motif both at the 5′ and 3′ termini (Supplementary Figure S2), suggesting an association of these three segments as a new tripartite virus. Due to its phylogenetic placement (Figure 5), we named it *Aspergillus flavus* vivivirus 1 (AfVV1). 

### 3.3. Transmission Electron Microscopy Observation of Viral Particles

**Figure 6:**
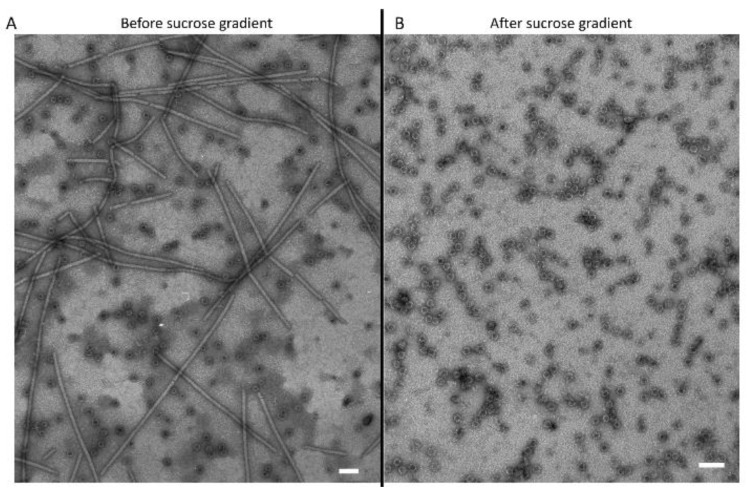
Transmission electron microscopy (TEM) imaging of virus particles isolated and purified from the MN1 strain. (**A**) Virus particle before the sucrose gradient separation, and (**B**) virus particles after a 50% to 20% sucrose gradient. Bar in each panel represents 100 nm length.

To associate AfVV1 to possible virions, a tentative viral purification was performed on liquid-grown mycelia of the MN1 strain. Interestingly, two different kinds of structure have been observed (Figure 6): a flexuous filament of about 13 nm in diameter and more than 1000 nm in length, most probably produced by *Aspergillus flavus* deltaflexivirus 1, and the second structure, revealed to be a small isometric and non-enveloped virus-like particle of about 14 nm in diameter, which was enriched after a further purification with a sucrose gradient, and which probably belongs to *Aspergillus flavus* vivivirus 1 (Figure 6B).

### 3.4. Correlation between AF Production and Viral Infection

A hypergeometric test was performed, and the result displayed that the expected number of virus-infected isolates producing AF would be 6.19, but in our case, we only obtained 1 isolate producing AF. Hence, the results are under-enriched by 6.19-fold compared to expectations, with a *p*-value of 8.5 × 10^−5^. Such result confirms that a correlation between viral infection and the inability of AF production is statistically supported.

## 4. Discussion

Due to its predominance amongst the fungal species that produce aflatoxins, and the derived relevance of the socio-economic issues correlated to the diffusion of mycotoxins on food and feed commodities all around the world, *A. flavus* is one of the most investigated species belonging to the *Aspergillus* genus. A plethora of studies have been performed around its physiology, ecology, and genetics [48,49,50]. Many aspects of the fungal morphogenesis have been elucidated through evidence obtained from *A. flavus*. For example, the role of methylation of specific sequences in the genomic DNA for the development of reproductive, dispersal, or resistance structures (vegetative spores and sclerotia), and the regulation of biosynthetic pathways for small bioactive molecules, critical for the interaction of Aspergilli with other organisms, that enable filamentous fungi to successfully exploit environmental resources by modifying the chemical diversity [51]. Recently, the complex ecological network of soil microbiota determining the niches that Aspergilli can fill-in was investigated, suggesting that interactions with the soil micro- and macro-biota deeply determine the role of secondary metabolite production to a great extent. However, it should be considered that selective forces maintaining the polymorphism of non-aflatoxigenic and aflatoxigenic colonies are mainly unknown. In this survey, different ratios of toxigenic/non-toxigenic and sclerotigenic/non-sclerotigenic were found. According to the literature, two aflatoxin chemotypes worldwide are often found to colonize the same local geographical areas [48], probably due to a dynamic, balancing selection that maintains genetic polymorphisms for aflatoxin production [49,50]. Additionally, the distribution of sclerotigenic strains observed within the *A. flavu*s population is consistent with data previously reported [52]. However, with both being dependent on secondary metabolisms, aflatoxin and perennation structures represent two faces of the same coin, which are thought to be related to a survival adaptation by influencing the persistence—or at least the fitness—of the organism in the environment.

Although attention has been paid to the environmental and cellular inputs driving the ecological features that characterize this species and its relationship with the environment, it is worth noting that very few studies looked at the role that mycoviruses can play in naturally occurring *A. flavus* isolates. As already well-established for the human virome, which has been correlated to various disease and physiological states [53,54,55,56], the identification and characterization of the mycovirome associated with a specific fungal population could provide a more detailed comprehension of the ecological relationships. It also represents, at the same time, an emergent tool to clarify some specific biological functions which could be linked to a viral infection and which can in turn impact both the fungal metabolism and the intra/interspecific interactions [10,57,58]. 

Here, we described for the first time the genome sequences of eight new mycoviral species infecting an *A. flavus* population isolated from a wide geographical region, and the virus-like particles associated with the recent newly reported group of viviviruses [42]. It is worth noting that the number of reports correlating the presence of mycoviruses in *A. flavus* with the production of aflatoxin is quite limited. This is remarkable in consideration of the average *Aspergillus* species infection rate, which accounts for about 10% of the isolates [59,60]. In contrast to existing literature, we observed that the percentage of virus-infected isolates resulted as doubled (more than 20%), suggesting a wider diffusion of mycoviruses from isolates that are maintained for a shorter time in axenic conditions than from isolates preserved for long time in mycological collection. As reviewed in 2017 [24], some mycovirus-infected *A. flavus* strains were reported [30,31,61,62], but the biological characterization and the possible impact on the fungal metabolic behavior were never assessed. More recently, a debilitation-associated partitivirus was reported in an isolate of *A. flavus* with a reduced conidiation and with an abnormal colony morphology [63]. In this case, the authors tried to characterize the impact of the viral infection on the pathogenic behavior but, unfortunately, despite the altered morphology in axenic conditions, it did not exhibit any decrease in virulence. 

Another important aspect of mycovirus infection is linked to the alteration of the primary and secondary metabolisms. An example of how mycoviruses can impact on fungal metabolisms is well-represented by the interaction between the model host *Cryphonectria parasitica* (the causal agent of chestnut canker) and its virus *Cryphonectria hypovirus 1*. In more detail, it was demonstrated that the mycovirus infection produce a wide metabolic reprogramming of its host, modifying the whole primary and secondary metabolisms [58,64]. The same results were also achieved in other species, where several studies demonstrated that mycoviruses are also able to modify the production of effectors and secondary metabolites [12,13,65]. Furthermore, two reference strains (NRRL 5565 and NRRL 5940) were studied because of their ability to produce aflatoxin and because they also harbor mycoviruses [30,31]. Initial studies on both of these isolates suggested that a mycoviral infection could impair aflatoxin production, but in a later study, no correlation between viral infection and mycotoxin production was reported [62]. Unfortunately, within the last 25 years, no additional studies concerning the identification of viruses infecting *A. flavus* and the possible correlation with aflatoxins’ production could be found. In this respect, our work provides a first insight of viral infections into a naturally occurring population coming from a large-scale sampling survey. Interestingly, we observed that, in our population, the infected isolates were predominantly non-toxigenic (only one aflatoxin-producing strain resulted positive to mycoviral infection, whereas the remaining 12 virus-infected isolates were not able to produce aflatoxin). On the other hand, it is worth nothing that, on the contrary to aflatoxins, the sclerotia production seems not to be correlated with the viral infection: among the 13 virus-infected isolates, 6 are unable to produce sclerotia, whereas 7 are sclerotia-producing isolates. Further screening on a wider population will clarify if any correlation exists between the aflatoxigenic behavior and the virological state.

**Figure 7:**
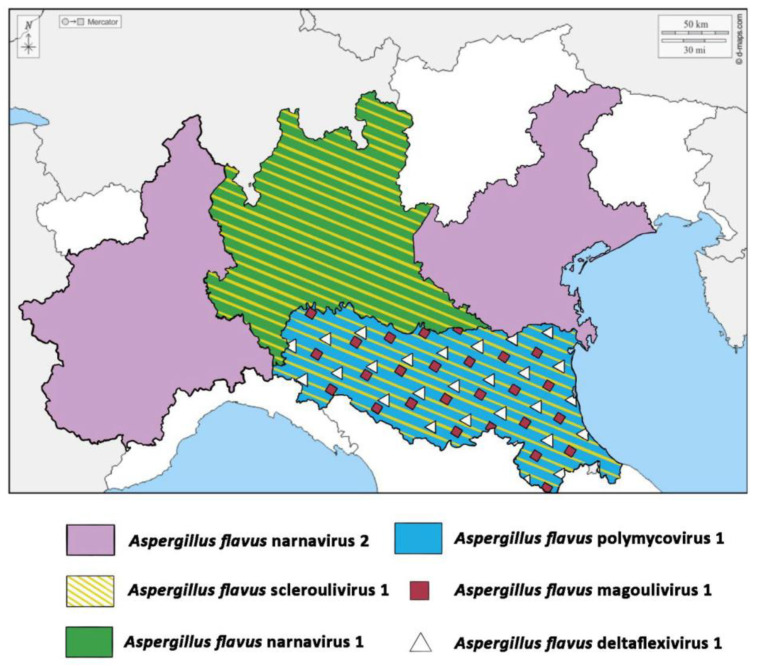
Map of Northern Italy showing the basin of origin of *A. flavus* strains isolated and used in the present work. Original map courtesy of: https://d-maps.com/carte.php?num_car=5892&lang=it, accessed on 22 May 2021.

From a geographical perspective, it is interesting to observe that viruses seem to be associated with specific regions (Figure 7). With the only exception of *Aspergillus flavus* narnavirus 2, that was present both in Piemonte and Veneto regions, which do not share borders, all the other viruses resulted strictly associated to the indigenous population of specific areas. For example, *Aspergillus flavus* partitivirus 2 was found only in isolates from Friuli-Venezia Giulia, *Aspergillus flavus* deltaflexivirus was found associated to the Emilia-Romagna region, whereas *Aspergillus flavus* narnavirus 1 is associated to isolates from Lombardia. These observations are interesting under an ecological perspective, pointing to an apparently limited movement of the fungal population also between bordering territories. This evidence suggests that mycoviruses could thus be exploited as molecular markers to be used for the monitoring of the fungal populations’ movement among territories, possibly representing an alternative to the use, in the future, of the more laborious molecular analyses currently applied for this purpose [66]. 

## 5. Conclusions

By providing a likely accurate description of the mycovirome present in an *A. flavus* population, representative of a wide geographical area such as the Po Valley, this survey offered an opportunity to reinterpret some biological standpoints in light of the virus–host interaction principles in *A. flavus*. These include the role of mycoviruses in the environmental inter- and intra-specific competition, including fungal development, their potential as a tool for biocontrol strategies against phytopathogenic/mycotoxigenic fungi, and as therapeutics [67]. The possibility to trace, for years to come, the linkage between the persistence of specific viruses and the niche colonization of different *A. flavus* populations within a specific geographic region is another interesting aspect. Our data pose new questions about the interaction between mycoviruses and aflatoxin production, suggesting a negative correlation that was previously hypothesized in old studies. More detailed research concerning the biological characterization of the mycoviruses described here will enhance the possibility to understand this correlation. Additionally, the significance of these mycoviruses to fungal ecology and viral evolution, and the potential for mycoviruses-based biological control of *A. flavus* infection, could largely benefit from the findings reported in the present work. Finally, the observed geographical correlation of some viral species would suggest the possibility to develop population studies using mycoviruses as biomarkers, highlighting both the interaction between compatible isolates and the movement of one isolate from one region to another. 

## Figures and Tables

**Table 1:** Biological characterization of *A. flavus* isolates in terms of aflatoxin production (AF) and sclerotia biogenesis and type (L: large sclerotia).

*A. flavus* Isolates		
Strain	AF	Sclerotia
CR1	-	L
CR5	-	L
CR8	-	-
CR9	-	L
Ven7	-	-
Pie6	-	-
Fri7	-	L
Fri3	yes	L
OZ7	-	L
OZ11	-	-
OZ15	-	L
Lom7	-	-
MN1	-	-
OZ1	yes	-
OZ2	yes	L
OZ3	yes	L
OZ6	yes	-
OZ8	yes	L
OZ16	yes	-
OZ19	yes	-
OZ4	-	L
OZ5	-	-
OZ9	-	L
OZ10	-	-
OZ14	-	L
OZ17	-	L
OZ18	-	-
Emi4	yes	L
Emi5	-	L
BO	yes	L
GR	yes	L
CSPT2	yes	L
CSPT7	-	L
Ven2	yes	L
Ven4	yes	-
Ven8	yes	L
Ven1	-	-
VRε	-	L
Fri2	yes	L
Fri6	yes	-
Fri8	yes	-
UD1	yes	-
UD4	yes	-
Fri5	-	-
UD3	-	-
AT1	yes	-
Lom6	yes	L
TNβ	yes	-
TNδ	yes	-
TNµ	yes	-
Pie10	yes	L
Pie1	-	L
Pie9	-	-
TOα	-	-
PRα	yes	-
CR6	yes	L
CR10	yes	L
CR16	yes	L
CR20	yes	-
CR18	yes	L
CR7	-	L
CR14	-	-

**Table 2:** Complete list of virus-infected *A. flavus* isolates with the hosted mycoviruses.

*A. flavus* Strain	Virus Detected
CR1	*Aspergillus flavus* polymycovirus 1
	*Aspergillus flavus* magoulivirus 1
CR5	*Aspergillus flavus* scleroulivirus 1
	*Aspergillus flavus* deltaflexivirus 1
CR8	*Aspergillus flavus* scleroulivirus 1
CR9	*Aspergillus flavus* polymycovirus 1
Ven7	*Aspergillus flavus* narnavirus 2
Pie 6	*Aspergillus flavus* narnavirus 2
Fri7	*Aspergillus flavus* partitivirus 2
Fri3	*Aspergillus flavus* partitivirus 2
OZ7	*Aspergillus flavus* deltaflexivirus 1
OZ15	*Aspergillus flavus* scleroulivirus 1 *Aspergillus flavus* deltaflexivirus 1
OZ11	*Aspergillus flavus* scleroulivirus 1
Lom7	*Aspergillus flavus* scleroulivirus 1
	*Aspergillus flavus* narnavirus 1
MN1	*Aspergillus flavus* narnavirus 1
	*Aspergillus flavus* vivivirus 1

**Table 3:** Summary of the new viruses detected in the present work: first hit in NCBI and putative function are reported for each genome segment, whereas genome type, viral group, and a putative name are reported for each new viral specie.

First Hit in NCBI		Identity	Putative Function	Viral Group	Proposed Name	Host (Isolate Acronym)
Accession	Organism					
QNQ74055.1	Plasmopara viticola lesion associated vivivirus 1	37.63%	RdRP	Unclassified	*Aspergillus flavus* vivivirus 1	CR9
QNQ74056.1	Plasmopara viticola lesion associated vivivirus 1	30.26%	Methyltransferase			
QDH90368.1	Riboviria sp.	48.86%	RdRP	Deltaflexivirus	*Aspergillus flavus* deltaflexivirus 1	CR5
QIR30284.1	Plasmopara viticola lesion associated narnavirus 5	63.68%	RdRP	Narnavirus	*Aspergillus flavus* narnavirus 1	Lom7, MN1
QIR30311.1	Plasmopara viticola lesion associated narnavirus 32	55.58%	RdRP	Narnavirus	*Aspergillus flavus* narnavirus 2	Ven7, Pie6
QQZ00864.1	Spodoptera exempta virus TenAfr-2017	48.49%	RdRP	Botourmiaviridae	*Aspergillus flavus* magoulivirus 1	CR1
QNN89181.1	Oidiodendron maius ourmia-like virus 1	43.31%	RdRP	Botourmiaviridae	*Aspergillus flavus* scleroulivirus 1	CR5, OZ15, OZ11, Lom7
APG78241.1	Hubei partiti-like virus 27	55.40%	RdRP	Partitiviridae	*Aspergillus flavus* partitivirus 2	Fri3, Fri7
BCD56394.1	Lichen partiti-like RNA virus sp.	34.22%	Coat protein			
AYP71801.1	Penicillium brevicompactum tetramycovirus 1	60.34%	RdRP	Polymycovirus	*Aspergillus flavus* polymycovirus 1	CR9
AYP71802.1	Penicillium brevicompactum tetramycovirus 1	54.63%	PAS-rich protein			
AYP71803.1	Penicillium brevicompactum tetramycovirus 1	50.82%	Methyltransferase			
AYP71804.1	Penicillium brevicompactum tetramycovirus 1	52.42%	Hypotetical protein			

## Data Availability

*A. flavus* strains used in this work are available upon request from the authors. The raw sequences used to assemble the viral genome are available in NCBI under accession number: SRR15015679. The mycoviral genomes are deposited in NCBI under the accession numbers: MZ600053 to MZ600068.

## Supplementary Materials

The following are available online at https://www.mdpi.com/article/10.3390/jof7100833/s1, Figure S1. Example of RAPD-PCR analysis output used for the molecular characterization of A. flavus isolates. The electrophoresis patterns of nine strains are reported. Figure S2. Alignments of the 5′ and 3′ UTR sequences of Aspergillus flavus vivivirus 1. On the left side the 5′ UTR of the three segments (RNA1, RNA2 and RNA3) were aligned using MUSCLE. On the right side the 3′ UTR of the three segments (RNA1, RNA2 and RNA3) were aligned using MUSCLE. Asterisks (*) denote the presence of the same nucleotide along the three segments. Table S1: List of primer pairs used in the present work to detect the new viral RdRPs.
