# Supplementary material for: Aspergillus Goes Viral: Ecological Insights from the Geographical Distribution of the Mycovirome within an Aspergillus flavus Population and Its Possible Correlation with Aflatoxin Biosynthesis
Source: J Fungi (Basel). 2021 Oct 5;7(10):833. doi: 10.3390/jof7100833 (PMC8538035; doi:10.3390/jof7100833)
Supplement: Supplementary file 1 [file jof-07-00833-s001.zip › jof-1357232-supplementary.pdf]

Supplementary Table S1. List of primer pairs used in the present work to detect the new viral RdRPs.

Primer name	Primer sequence
Tub-1	Fw: 5'TACCATGGACGCCGTCCG3'<br>Rev: 5'GACGGACAACATCGACAAC3'
Aspergillus flavus polymycovirus 1	Fw: 5'CGGCGTGCTCTGGACTCCTG3'<br>Rev: 5'TCACACATCATGGCGCG3'
Aspergillus flavus magoulivirus 1	Fw: 5'TCTTAACCCAGCCCGCCGTG3'<br>Rev: 5'GTTTTGCGAGACGAGGCGGT3'
Aspergillus flavus scleroulivirus 2	Fw: 5 GGGCTGGAGGGGCGAAACAA3'<br>Rev: 5'GGTCGGCGGGATCTTCGGAC3'
Aspergillus flavus deltaflexivirus 1	Fw: 5'CGTCTGGAGAGGGACCCGGT3'<br>Rev: 5'CCGCCCCTCAACCCCTTACG3'
Aspergillus flavus narnavirus 1	Fw: 5'AACAGCCCCTTCCAAGCCGG3'<br>Rev: 5'GCCTCTCCCCGTTACGCAGG3'
Aspergillus flavus narnavirus 2	Fw: 5'GTCGCCTGCTCCCAATCCGT3'<br>Rev: 5'GTGCCGCTAATCACGCTTGGA3'
Aspergillus flavus vivivirus 1	Fw: 5'GGTGGCTAACTGGTCGCGGT3'<br>Rev: 5'GTACCGCCGGCCATTCGAGA3'
Aspergillus flavus partitivirus 2	Fw: 5'AAACAGATTATCACGGACGAGATG3'<br>Rev: 5'TCCTCATGTGATTCGACCAGAT3'

Supplementary Figure S1. Example of RAPD-PCR analysis output used for the molecular characterization of *A. flavus* isolates. The electrophoresis patterns of nine strains are reported.

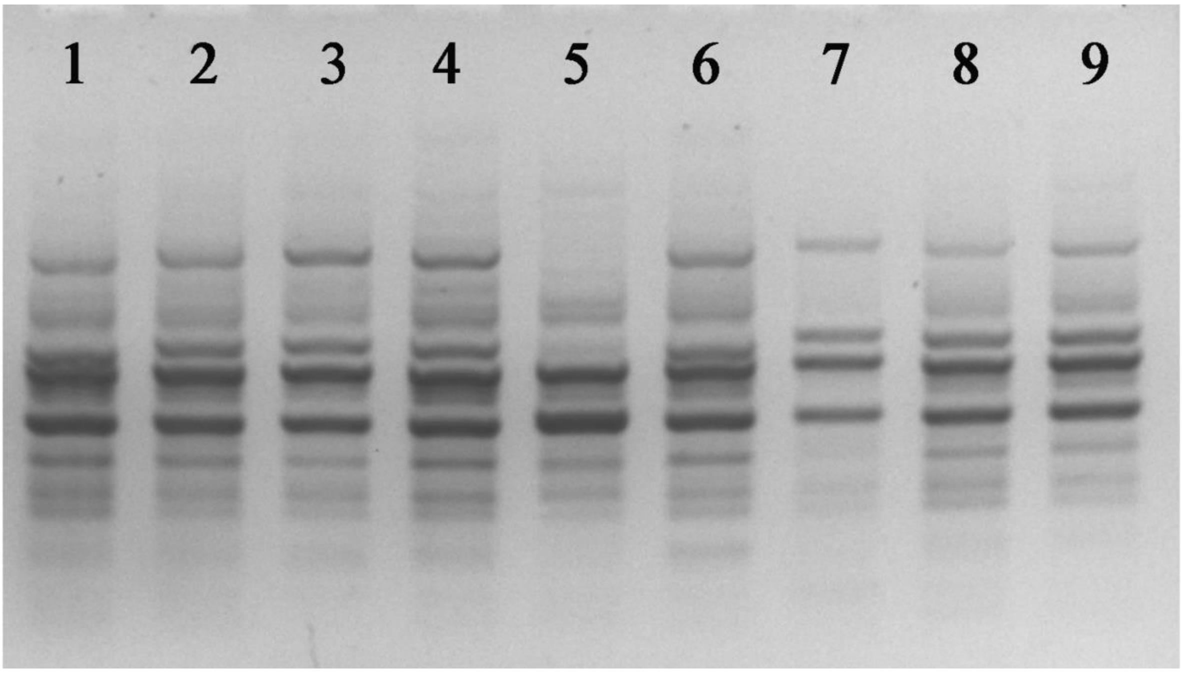

Supplementary Figure S2. Alignments of the 5' and 3' UTR sequences of *Aspergillus flavus* vivivirus 1. On the left side the 5' UTR of the three segments (RNA1, RNA2 and RNA3) were aligned using MUSCLE. On the right side the 3' UTR of the three segments (RNA1, RNA2 and RNA3) were aligned using MUSCLE. Asterisks (*) denote the presence of the same nucleotide along the three segments.

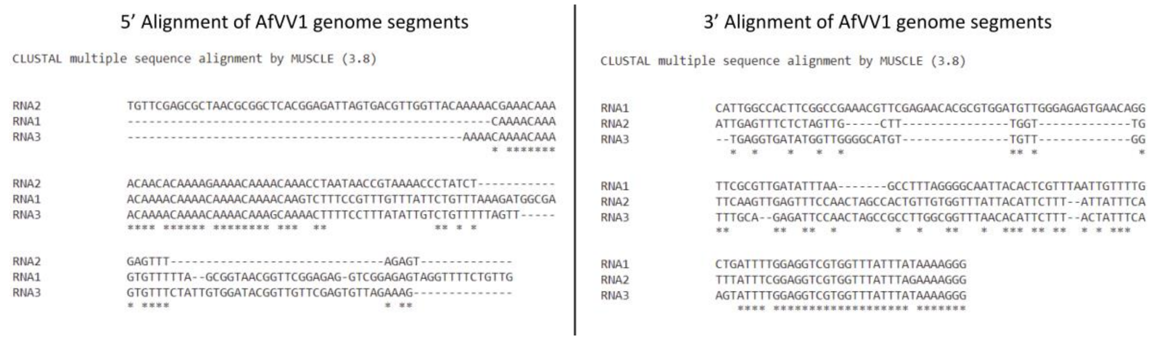

5' Alignment of AfVV1 genome segments

```
CLUSTAL multiple sequence alignment by MUSCLE (3.8)


RNA2            TGTTCGAGCGCTAACGCGGCTCACGGAGATTAGTGACGTTGGTTACAAAAACGAAACAAA
RNA1            ---------------------------------------------------CAAAACAAA
RNA3            -----------------------------------------------AAAACAAAACAAA
                                                                  * *******

RNA2            ACAACACAAAAGAAAACAAAACAAACCTAATAACCGTAAAACCCTATCT-----------
RNA1            ACAAAACAAAACAAAACAAAACAAGTCTTTCCGTTTGTTTATTCTGTTTAAAGATGGCGA
RNA3            ACAAAACAAAACAAAACAAAGCAAAACTTTTCCTTTATATTGTCTGTTTTTAGTT-----
                **** ****** ******** ***  **               ** * *

RNA2            GAGTTT------------------------------AGAGT-------------
RNA1            GTGTTTTTA--GCGGTAACGGTTCGGAGAG-GTCGGAGAGTAGGTTTTCTGTTG
RNA3            GTGTTTCTATTGTGGATACGGTTGTTCGAGTGTTAGAAAG--------------
                * ****                              * **
```

3' Alignment of AfVV1 genome segments

```
CLUSTAL multiple sequence alignment by MUSCLE (3.8)


RNA1            CATTGGCCACTTCGGCCGAAACGTTCGAGAACACGCGTGGATGTTGGGAGAGTGAACAGG
RNA2            ATTGAGTTTCTCTAGTTG-----CTT---------------TGGT-------------TG
RNA3            --TGAGGTGATATGGTTGGGGCATGT---------------TGTT-------------GG
                  *  *    *   *  *                      ** *              *

RNA1            TTCGCGTTGATATTTAA-------GCCTTTAGGGGCAATTACACTCGTTTAATTGTTTTG
RNA2            TTCAAGTTGAGTTTCCAACTAGCCACTGTTGTGGTTTATTACATTCTTT--ATTATTTCA
RNA3            TTTGCA--GAGATTCCAACTAGCCGCCTTGGCGGTTTAACACATTCTTT--ACTATTTCA
                **      **  **  *         *  *   **    *  *** ** **  * * ***

RNA1            CTGATTTTGGAGGTCGTGGTTTATTTATAAAAGGG
RNA2            TTTATTTCGGAGGTCGTGGTTTATTTAGAAAAGGG
RNA3            AGTATTTTGGAGGTCGTGGTTTATTTATAAAAGGG
                   **** ****************** *******
```
